# Cervical cancer treatment costs and cost-effectiveness analysis of human papillomavirus vaccination in Vietnam: a PRIME modeling study

**DOI:** 10.1186/s12913-017-2297-x

**Published:** 2017-05-15

**Authors:** Hoang Van Minh, Nguyen Thi Tuyet My, Mark Jit

**Affiliations:** 1 Hanoi University of Public Health, Building A, 1A Duc Thang Road, Duc Thang Ward, North Tu Liem District, Hanoi, Vietnam; 2 Department of Infectious Disease Epidemiology, London School of Hygiene and Tropical Medicine, London, UK; 3 Modelling and Economics Unit, Public Health England, London, UK

**Keywords:** Cost-effectiveness analysis, Cervical cancer, Vietnam, PRIME, Human papillomavirus

## Abstract

**Background:**

Cervical cancer is currently the leading cause of cancer mortality among women in South Vietnam and the second leading cause of cancer mortality in North Vietnam. Human papillomavirus (HPV) vaccination has the potential to substantially decrease this burden. The World Health Organization (WHO) recommends that a cost-effectiveness analysis of HPV vaccination is conducted before nationwide introduction.

**Methods:**

The Papillomavirus Rapid Interface for Modeling and Economics (PRIME) model was used to evaluate the cost-effectiveness of HPV vaccine introduction. A costing study based on expert panel discussions, interviews and hospital case note reviews was conducted to explore the cost of cervical cancer care.

**Results:**

The cost of cervical cancer treatment ranged from US$368 – 11400 depending on the type of hospital and treatment involved. Under Gavi-negotiated prices of US$4.55, HPV vaccination is likely to be very cost-effective with an incremental cost per disability-adjusted life year (DALY) averted in the range US$780 - 1120. However, under list prices for Cervarix and Gardasil in Vietnam, the incremental cost per DALY averted for HPV vaccination can exceed US$8000.

**Conclusion:**

HPV vaccine introduction appears to be economically attractive only if Vietnam is able to procure the vaccine at Gavi prices. This highlights the importance of initiating a nationwide vaccination programme while such prices are still available.

## Background

Infection with human papillomavirus (HPV) is a cause of many diseases including cervical cancer. About 90 types of human papillomavirus have been found but two types (16 and 18) are responsible for 75% to 80% of cases of cervical cancer in Europe and North America [1], and more than 70% of cases in Asia [2]. In 2012, cervical cancer was the fourth most common type of cancer among women worldwide with an estimated 528,000 new cases and 266,000 deaths; 87% of deaths occurred in low and middle-income countries [3]. Treatment costs for cervical cancer remain high with low survival probabilities due to late diagnosis in many countries [4, 5]. HPV vaccines offer an approach for primary prevention of HPV infection that can eventually reduce the health and economic burden of cervical cancer. Cost-effectiveness studies in many countries using a range of model types have suggested that vaccinating young adolescent females can be cost-effective [6–12].

In Vietnam, there are no comprehensive national figures on cervical cancer incidence. However, studies based on smaller samples from older registry-based studies have found that incidence ranged from 6.8 cases per 100,000 women in the north of Vietnam [13] to 26 cases per 100,000 women in the south of Vietnam [14]. As estimated, cervical cancer is the main cause of female cancer mortality Ho Chi Minh City and the second main cause in Hanoi [15].

Vietnam began its accelerated transition policy from support from Gavi, the Vaccine Alliance, in 2016 [16]. While the country is no longer eligible for Gavi support, there is a window of opportunity where is can still procure vaccines at Gavi prices [17]. The Vietnam National Institute of Hygiene and Epidemiology (NIHE) remains keen on introducing additional vaccines despite tight resource constraints. Vietnam has not yet incorporated HPV vaccines into its routine vaccination schedule, although pilot studies have been conducted in the country [18]. A previous cost-effectiveness analysis concluded that HPV vaccination combined with regular screening could be cost-effective provided that vaccine prices were low (below I$25 per vaccinated girl) [19]. However, there has yet to be a cost-effectiveness study on HPV vaccination involving local analysts and stakeholders. There has also not been a study estimating the cost of cervical cancer and pre-cancer treatment in Vietnam, as the previous study extrapolated costing data from Thailand and other countries. Hence we aimed to perform the first local cost-effectiveness analysis of human papillomavirus vaccines in Vietnam using the Papillomavirus Rapid Interface for Modeling and Economics (PRIME).

## Methods

### Mathematical model

We used PRIME, a static model supported by the World Health Organization to facilitate estimating the cost-effectiveness of HPV vaccine introduction in order to support decisions about vaccine introduction, particularly in low and middle-income countries. An Excel-based interface to PRIME is available in the public domain (www.primetool.org) for country studies. This consists of a number of spreadsheets to input country-level data on demography, cervical cancer incidence and mortality, and costs. Outputs can be visualised in terms of tables and graphs. The Excel-based version of the model can be used to evaluate the cost-effectiveness of vaccinating females prior to sexual debut. Because it excludes herd effects and details of cancer natural history, it is not designed to evaluate other options such as vaccinating males, vaccinating older females, catch-up campaigns or changes to cervical cancer screening. The detailed model structure and assumptions have been previously described [20].

### Methodological assumptions

Methodological assumptions used in the analysis followed WHO guidelines for cost-effectiveness evaluations [21]. In particular, we adopted a a discount rate of 3% for both costs and health effects on a lifetime time horizon. However, a health care provider perspective was adopted because of the unavailability of household expenses for cervical cancer patients in Vietnam. Health outcomes were measured in disability-adjusted life years (DALYs) rather than quality-adjusted life years (QALYs) because quality of life weights associated with cervical cancer have never been elicited from a Vietnamese population.

PRIME’s default data (obtained from publicly available databases and the literature) were used to inform parameters related to vaccine efficacy, population demography and cervical cancer incidence [20]. However, costs for vaccine delivery and cervical cancer treatment were estimated from primary sources.

### Vaccination assumptions

School-based HPV vaccine demonstration projects organized by the Program for Appropriate Technology in Health (PATH) in Vietnam among 11 year olds achieved coverage of 96.1% [22]. Hence we assumed that vaccination would be delivered to 11 year olds and achieve 95% coverage. The current HPV vaccine schedule recommended by NIHE is three doses.

### Vaccine delivery costs

We conducted a costing study which used a bottom-up approach to calculate the financial cost of HPV vaccine delivery from central to commune level (i.e. between NIHE in Hanoi, the provincial centers for preventive medicine, the district health centers, and the commune health station), incorporating both capital costs and recurrent costs. We also assumed minimal wastage of vaccine doses, due to annual training of vaccine delivery staff and use of single-dose vials in the Vietnamese national immunization program.

Financial costs estimated included both start-up and recurrent costs. Start-up costs were defined as initial investments whose benefits last longer than one year such as initial training, social mobilization, information, education and communication material development, cold chain equipment purchase and vehicles purchase. Recurrent costs were defined as the cost of resources whose benefits last one year or less such as the personnel time, transport, maintenance, monitoring and evaluation, and supervision as well as costs of short-term training activities that lasted a year or less.

Costs for healthcare personnel for each activity was estimated based on their time and monthly salary. To collect information related to these personnel costs, one staff member at each health facility was interviewed and monthly salary reports were collected. For start-up costs, we assumed a discount rate of 3% and useful lifecycle of 5-10 years.

### Treatment costs

To estimate the cost of pre-cancer and cancer treatment, we conducted a study involving clinician and patient interviews, and hospital record reviews. This consisted of two phases.

Hospitals and health centers in Vietnam are stratified into four levels: central, provincial, district and commune. While provincial and district hospitals provide some treatment of pre-cancerous neoplasias, cervical cancer treatment is only provided at the central level. We randomly selected two central hospitals (Ho Chi Minh City Cancer Hospital and Hue Central Hospital) from a list of all hospitals in Ho Chi Minh City and Hue.

In phase I of the study, we invited healthcare workers from the selected hospitals to construct scenarios that represent patient treatment situations (see Table 1). In these hospitals, 40 staff from different departments (including examination, laboratory medicine, radiotherapy and chemotherapy) participated.

**Table 1:** Scenarios describing hospitalized cervical cancer patients for costing by the expert panel

Scenario	Hospital level	Diagnosis	Type of treatment	Length of treatment (days)	Number of days of productivity loss
Scenario 1	Central, provincial and district	CIN I	Annual screening	1	1
Scenario 2	Central, provincial and district	CIN I	Cryo therapy	2	3
Scenario 3	Central and provincial	CIN II & III	LEEP	4	5
Scenario 4	Central and provincial	CIN II &III	Conization	4	5
Scenario 5	At central level	IA1 - IIB proximal	Radical hysterectomy	7	7
Scenario 6	At central level		Radical hysterectomy + Chemoradiation	30	150
Scenario 7	At central level		Radical hysterectomy + Radio therapy (Intr)	30	150
Scenario 8	At central level		Radical hysterectomy + Radio-therapy (External beam)	30	150
Scenario 9	At central level	IIB distant - IVB	Chemoradiation	30	150
Scenario 10	At central level		Radio therapy (Intra + External)	30	150
Scenario 11	Average cost of scenario 5 to 10				

In phase II, we micro-costed each of the scenarios identified by interviewing the healthcare workers in detail. In addition, we reviewed medical records and financial bills by randomly selecting 38 patients in the selected hospitals to extract information related to cancer stage, patient age, diagnosis, treatment and length of treatment. Further to that, we interviewed the 38 patients and their family members to get detailed costing information.

### Sensitivity analysis

Model uncertainty was explored by varying parameters governing vaccine delivery and treatment costs according to the scenarios above. Vaccine prices were also varied between the Gavi negotiated price of United States Dollar (USD) 4.55 per dose, the market price of Cervarix in Vietnam of USD 35.60 per dose and the market price of Gardasil in Vietnam of USD 55.80 per dose.

## Results

Table 2 shows the parameters used in the model, including birth cohort size, coverage of 3 vaccine doses, vaccine efficacy vs HPV 16/18, vaccine price per fully immunized girl, vaccine delivery cost per fully immunized girl, cancer treatment cost as per patient scenario, DALYs incurred due to non-fatal and fatal cancer episodes, and epidemiological data such as cervical cancer incidence and mortality. The female birth cohort size was 556,874 in 2014. Vaccine price per fully immunized girl was estimated to be USD 15-168. Cancer treatment cost for 10 patient scenarios ranged from USD 368-11448 depending on the kind of treatment. Information related to cervical cancer incidence and mortality was extracted from GLOBOCAN 2012. All-cause mortality in 2012 was extracted from national data.

**Table 2:** Selected parameters for estimating cost-effectiveness of HPV vaccination using the PRIME model

No	Parameter	Average value/value	Range	Source
1	Vaccine delivery cost per FIG	$ 10.4	$ 9.5-$ 11.3	Costing study
2	Cancer treatment cost			Costing study
	Scenario 5 (IA1 - IIB proximal treated by Radical hysterectomy)	$ 368.3	N/A	
	Scenario 6 (IA1 - IIB proximal treated by Radical hysterectomy + Chemoradiation)	$ 8700.1	N/A	
	Scenario 7 (IA1 - IIB proximal treated by Radical hysterectomy + Radio therapy (Intr))	$ 2209.6	N/A	
	Scenario 8 (IA1 - IIB proximal treated by Radical hysterectomy + Radio-therapy (External beam))	$ 1449.9	N/A	
	Scenario 9 (IIB distant – IVB treated by Chemoradiation)	$ 11448.1	N/A	
	Scenario 10 (IIB distant – IVB treated by Radio therapy (Intra + External))	$ 5166.52	N/A	
	Scenario 11 (average cost of all scenarios)	$ 4,890.44	N/A	
3	DALYs lost per non-fatal cancer episode	0.7		Literature review
4	DALYs lost per fatal cancer episode (excluding life years lost)	0.8		Literature review
5	Epidemiological data			Literature review
	Cervical cancer incidence in 2012	6.1 per 100000		GLOBOCAN 2012
		20.5 per 100000		
		26.7 per 100000		
		31.3 per 100000		
		32.1 per 100000		
		26.2 per 100000		
		20.5 per 100000		
		13 per 100000		
	Cervical cancer mortality in 2012	1.1 per 100000		GLOBOCAN 2012
		6.5 per 100000		
		15.1 per 100000		
		18.8 per 100000		
		21.3 per 100000		
		21.8 per 100000		
		20 per 100000		
		16.1 per 100000		
		5.3 per 100000		
	All-cause mortality in 2012	100 - 52 800 per 100 000 (depending on age)		National statistics

Table 3 compares the cost-effectiveness of HPV vaccination in Vietnam under different assumptions about the cost of cervical cancer treatment (depending on the type of treatment and stage of cancer) and the cost of vaccination. If HPV vaccines can be procured at prices negotiated by Gavi, then the incremental cost per DALY averted ranges between USD18 (under scenario 9) - USD1,103 (under scenario 5). However, if either vaccine is only available at list prices, then the incremental cost per DALY averted increases dramatically, exceeding USD4,000 in all the scenarios explored. Using the average cost of cancer treatment over the six scenarios gives an incremental cost per DALY of USD800 at Gavi prices, and over USD5,000 at list prices.

**Table 3:** Estimation of incremental costs as per life saved, per life year saved and per DALY averted in the PRIME

Estimation of incremental costs	Vaccine price	Scenario 5	Scenario 6	Scenario 7	Scenario 8	Scenario 9	Scenario 10	Scenario 11 (average cost)
Incremental cost per discounted life saved	HPV vaccine with negotiated price from GAVI (13.65 US$ for 3 doses)	24,490	5,773	20,354	22,061	401	13,711	14,331
	Cervarix vaccine with 106.8 US$ for 3 doses	122,510	103,793	118,374	120,081	97,619	111,731	112,351
	Gardasil vaccine with 167.4 US$ for 3 doses	186,279	167,561	182,142	183,849	161,388	175,499	176,120
Incremental cost per discounted life year saved	HPV vaccine with negotiated price from GAVI (13.65 US$ for 3 doses)	1,196	282	994	1,077	20	670	700
	Cervarix vaccine with 106.8 US$ for 3 doses	5,983	5,069	5,781	5,865	4,768	5,457	5,487
	Gardasil vaccine with 167.4 US$ for 3 doses	9,098	8,184	8,896	8,979	7,882	8,571	8,602
Incremental cost per discounted DALY averted	HPV vaccine with negotiated price from GAVI (13.65 US$ for 3 doses)	1,103	260	917	994	18	618	645
	Cervarix vaccine with 106.8 US$ for 3 doses	5,518	4,675	5,331	5,408	4,397	5,032	5,060
	Gardasil vaccine with 167.4 US$ for 3 doses	8,390	7,547	8,203	8,280	7,269	7,904	7,932

Unit cost: US$

## Discussion

Our analysis suggests that routine HPV vaccination of 11-year-old girls in Vietnam has an incremental cost-effectiveness ratio of around USD780 - 1200 per DALY averted and USD7000 - 23000 per life saved, if the vaccine can be procured at prices available to Gavi, the Vaccine Alliance. This is well below Vietnam’s Gross Domestic Product (GDP) per capita, and hence at the level commonly considered “very cost-effective” based on global recommendations in WHO-CHOICE.

There are large disparities in cervical cancer incidence between north and south Vietnam, so the cost-effectiveness of vaccination is likely to differ between regions. However, we have performed our analysis on a national level because the most recent cervical cancer incidence data is only available at the national-level, and because a national program is likely to be more acceptable to policy makers.

More recently, the use of GDP per capita-based thresholds for cost-effectiveness has been criticized [25] and is no longer recommended by WHO. Several alternatives have been suggested, including comparing the incremental cost-effectiveness of new interventions to existing interventions, and measuring the willingness to pay for health improvements. In terms of the first approach, the cost per disability-adjusted life year (DALY) averted of HPV vaccination is similar to previously published figures for rotavirus (USD540 per DALY averted) [23], and slightly lower than figures for Haemophilus influenza type b (USD1373 per DALY averted) [24]. The cost per life saved is within the range of estimates for corresponding figures for existing vaccines in Vietnam’s Expanded Programme on Immunization (USD1000 - 27,000 per life saved) [26, 27]. For the second approach, the only relevant study to refer to is one in a rural area of Vietnam in 2014. This revealed that respondents were willing to pay USD667 - 992 for a quality-adjusted life year (QALY) [28]. The results would likely be higher if urban respondents were surveyed. Nevertheless, the results suggest that HPV vaccination is likely to be cost-effective in Vietnam if the country is able to access Gavi prices, but the cost-effectiveness would probably not be favourable if higher prices had to be paid.

A study conducted to assess Vietnamese mothers’ willingness to pay for HPV vaccination for their daughters suggested that nearly 70% of them would pay for HPV vaccines at $7 per course, but this drops to 30% at $353 per course [29]. This suggests that even at Gavi-negotiated prices, public procurement of HPV vaccines is necessary in order to see substantial vaccine uptake in the population.

If Vietnam is not eligible to purchase vaccine prices at Gavi-negotiated levels, then vaccination appears much less cost-effective than other vaccines, and its incremental cost-effectiveness ratio well exceeds GDP per capita. This highlights the value of pooled procurement mechanisms such as those used by Gavi and the Pan-American Health Organization (PAHO) in obtaining fair prices in low- and lower-middle income countries [30]. It also underscores the urgency of HPV vaccine introduction in Gavi-graduating countries like Vietnam while they are still eligible for lower vaccine prices. Analyses such as these are also helpful to local policy makers for negotiating long-term prices with vaccine manufacturers. Further analyses could take into account constraints of both cost-effectiveness and affordability in different regions and socio-economic contexts in Vietnam.

Only one previous cost-effectiveness analysis of HPV vaccination in Vietnam has been conducted [19], which used an individual-based Monte Carlo simulation model developed at Harvard University. Like our analysis, the Harvard analysis concluded that HPV vaccination would only be cost-effective at low vaccine prices (below about $10 per vaccinated girl in north Vietnam and $50 in south Vietnam). The advantage of our study is that we were able to use actual vaccine prices available to Gavi and on the private market in Vietnam. We were also able to micro-cost cervical cancer treatment costs for the first time, instead of extrapolating costs from Thailand and other Asian countries as was done in the Harvard study.

Vietnam is one of the pioneer countries to use PRIME with WHO support to inform a national cost-effectiveness analysis of HPV vaccination. The tool was found by local analysts to have both positive features and shortcomings. Data and expertise requirements are light, facilitating its use and interpretation by non-experts. This allowed analyses to be led by local investigators in contrast to many previous cost-effectiveness analyses in low and middle-income countries [31]. However, PRIME underestimates the cost-effectiveness of HPV vaccination by not incorporating herd protection, pre-cancerous health states or non-cervical cancer disease outcomes such as anogenital warts. Also, the Excel-based version lacks built-in features for probabilistic sensitivity analysis, so outputs such as cost-effectiveness acceptability curves have to be constructed based on manual alteration of input parameters. Hence there is scope for future iterations of PRIME to incorporate more advanced features for expert users.

## Conclusion

This is the first cost-effectiveness analysis of HPV vaccination in Vietnam to be led by local analysts and to include a local costing study of cervical cancer treatment. The study highlights the urgency of HPV vaccine implementation in Vietnam during the window of opportunity to obtain Gavi prices. It also demonstrates the importance of HPV vaccine introduction in Gavi-graduating countries before the countries become ineligible for low Gavi prices, after which the vaccination programme may cease to be cost-effective. Finally, our study highlights the need to understand realistic cost-effectiveness thresholds in low and middle-income countries so as to avoid misinforming health policy makers.

## Abbreviations

DALY: Disability-adjusted life years
GDP: Gross domestic product
HPV: Human papillomavirus
NIHE: National Institute of Hygiene and Epidemiology
PAHO: Pan-American Health Organization
PATH: Program for Appropriate Technology in Health
PRIME: Papillomavirus Rapid Interface for Modelling and Economics (PRIME)
QALY: Quality-adjusted life years
USD: United States dollar
WHO-CHOICE: World Health Organization CHOosing Interventions that are Cost-Effective initiative
